# Ameliorative effect of apelin-13 against renal complications in L-NAME-induced preeclampsia in rats

**DOI:** 10.7717/peerj.11110

**Published:** 2021-03-31

**Authors:** Reham Z. Hamza, Abdel Aziz A. Diab, Mansour H. Zahra, Ali K. Asalah, Mai S. Attia, Suzan MM Moursi

**Affiliations:** 1 Biology Department, College of Sciences, Taif University, Taif, Taif, Saudi Arabia; 2 Zoology Department, Faculty of Science, Zagazig University, Zagazig, Egypt; 3 Medical Physiology Department - Faculty of Human Medicine, Zagazig University, Zagazig, Egypt

**Keywords:** Apelin, Preeclampsia, Proteinuria, Kidney injury

## Abstract

Pre-eclampsia (PE) accompanying acute liver and kidney injury has remained a master cause of both fetal and maternal mortality and morbidity. Vasoactive mediators, oxidative stress and inflammatory imbalanceshave an important role in PE pathogenesis. Apelin is an adipokine that improves endothelial dysfunction; has anti-inflammatory and antioxidant effects; moreover, its level reduced during PE. This study aimed to explore the effects of apelin-13 administration on preeclampsia-associated renal dysfunction and proteinuria. Thirty-three pregnant female rats were divided into three groups; group: 1 (normal pregnant rats), group: 2 (preeclamptic rats); where rats were injected subcutaneously with 75 mg L-NAME/ kg body weight/day beginning from 9th to 20th day of pregnancy andgroup 3 (apelin-13 treated preeclamptic rats); In which L-NAME-induced preeclamptic rats were subcutaneously injected with 6 × 10^−8^ mol apelin-13/kg body weight/twice daily starting from 6th to 20th day of pregnancy. In all groups, mean arterial blood pressure, total urine protein, serum urea, creatinine, nitric oxide (NO), endothelin-1 (ET-1), interleukin–6 (IL-6) and malondialdhyde (MDA) were measured. Histopathological examination of kidney tissues was also done. preeclamptic rats showed significantly increased mean arterial blood pressure, total urine proteins, serum urea, creatinine, ET-1, IL-6, and MDA, but revealed a significantly decreased serum NO level. On the other hand, apelin treatment significantly improved these parameters together with amelioration of kidney histoarchitecture in the treated group. In conclusion, apelin may be a potentially curative candidate for prohibiting kidney damage and have a therapeutic benefit in PE rat models.

## Introduction

Pre-eclampsia (PE) is a miscellaneous and multi-organ disturbance distinguished by extensive dysfunctions such as placental ischemia, vascular injury and the severe damage on kidney and liver (Pettit et al., 2015). The whole kidney texture might be influenced, resulting in acute kidney injury (AKI), and other pregnancy-related harms, such as shock and sepsis (Villie et al., 2018).

Elevated proteinuria might turn nephrotic at an early stage during the pregnancy, with an increased danger of intravascular volume reduction, thrombosis, reflux nephropathy and patients are at a high risk of promoting urinary tract infections through this interval (Mehrabadi et al., 2014).

Throughout normal pregnancy, the blood volume, cardiac output and heart rate are elevated; but, the blood pressure (BP) yet normal or is lower than that before the pregnancy. Nitric oxide (NO) has a remarkable role in the organization of the cardiovascular system through pregnancy (European Society of Gynecology, 2011).

Pre-eclampsia is resulted from irregular placentation with changes in renin/angiotensin/aldosterone system and angiogenic proteins, causing endothelial damage, hypertension and organ dysfunction (Hussein & Lafayette, 2014a; Hussein & Lafayette, 2014b). PE can be induced by L-NAME, a recognized inhibitor of NO production. When it is administered in animals during their middle to late period of gestation, a syndrome resembling preeclampsia in human is induced, which is manifested by diminution of perfusion of placenta, elevated reactive oxygen species (ROS) production, hypertension and proteinuria (Sedeek et al., 2008).

Methyldopa and hydralazine are the two most prescribed agents for PE. These drugs decrease blood pressure, but they are not reno-protective and do not have any effect on proteinuria. Moreover, angiotensin inhibitors (the main antihypertensive drugs with reno-protective property) are contraindicated in pregnancy. Thus, it is necessary to evolve safe and efficacious drugs to ameliorate proteinuria and AKI in PE (Talebianpoor et al., 2017).

Interestingly, apelin decreased in both placental chorionic villi and serum of patients with PE (Taha, Zahraei & Al-Hakeim, 2020), and although a high homology between the angiotensin II receptor type 1(AT-1) receptors and the G protein-coupled apelin receptor (APJ) and the tissue expression for both receptors are similar, contrasting actions between apelin/APJ system and the angiotensin II /AT-1 system have been exhibited (Fukushima et al., 2010).  

Moreover, the apelin/APJ system may have protective roles in various kidney diseases. It helps tissue perfusion and produces antifibrotic tissue-safeguarding effects in ureteral obstruction as a type of renal fibrosis (Nishida et al., 2012). It has also been reported that apelin inhibited renal dysfunction in adenine-induced chronic renal failure in rats (Abul-fadle, Saied & Musa, 2018) and podocyte autophagy in diabetes (Liu, Zhang & Wang, 2017). Furthermore, apelin ameliorated the affected renal function and histological structure, decreased inflammatory mediators and ROS, and hampered cell apoptosis in renal ischemia/reperfusion injury (Bircan et al., 2016 and Zhang et al., 2020).

Considering the above data implying the protective role of apelin in kidney diseases and given the multiple effects of apelin on vasodilation, angiogenesis and inhibition of the oxidative stress and inflammation (Wang et al., 2017), it is reasonable to speculate that apelin may ameliorate the pathogenesis of preeclampsia associated renal affection.

Therefore, we designed this project to explore the possible implications of apelin-13 on kidney functions in L-NAME induced rat model of preeclampsia and to distinguish the potential involved processes.

## Material and Methods

This research was done in faculty of medicine, zagazig university and involved 50 adult albino rats (40 female rats weighing 119–140 g, 50 day old and 10 adult male rats for fertilization weighing 210–238 g), were purchased from faculty of veterinary medicine- zagazig university. Rats were retained in metal cages under hygienic conditions, had free access to food and water, and kept at room temperature on a 12 h light and 12 h dark. All the experimental methodology has been managed in accordance with the guiding standard for the sponsor and use of research animals approved and reviewed by ZU-IACUC committee, with number ZU-IACUC/ 1/ F/ 78/ 2019.

### Induction of pregnancy

Rats were checked for estrous cycles for 2 sequential weeks. Non-stained vaginal excretions were directly examined each morning under a light microscope (40x) and the phases of the estrus cycle were detected by investigating the vaginal cytology. The estrus phase was determined by increasing the number of cornified or irregular shaped epithelial cells in the vaginal smear.

Female rats in estrous stage were placed with male rats in a separate enclosure. In the next morning, female rats were separated to confirm copulation by the existence of sperms within the vaginal smear. The existence of sperms indicates the 1st day of gestation (Abdul Aziz et al., 2016). seven rats from the forty did not get pregnant and have been excluded from the study. Therefore, 33 pregnant rats were used in the experiment.

### Experimental groups

Thirty three (33) pregnant rats were divided into 3 groups:

**Group 1** (normal pregnant group; *n* = 10) has been injected subcutaneously (s.c) with saline as placebo from 9th to 20th day of gestation.

**Group 2** (PE-induced group; *n* = 12) rats were injected s.c with N-nitro-L-arginine methyl ester (L-NAME) (obtained from Sigma Aldrich Co., USA) at a dose of 75 mg/kg B.wt/daily beginning from 9th to 20thday of gestation (Shu et al., 2018).

**Group 3 (**PE-induced group supplemented with apelin; *n* = 11) rats received L-NAME as previously described in the previous group and simultaneously injected s.c with apelin-13 (obtained from Sigma Aldrich Co., USA) at a dose of 6 × 10^−8^ mol/ kg/ twice daily beginning from 6thto 20thday of gestation (Wang et al., 2017)  to allow it to start its protective effects before L-NAME administration while the other groups simultaneously received saline.

### Measurement of blood pressure

Systolic, diastolic and mean arterial BP were measured on 18th day pregnancy, using noninvasive BP measurement system (BIOPAC system, Inc.; USA) (Abubakar, Ukwuani & Mande, 2015).

### Urine collection

The 24 h urine samples were collected at gestational day (GD)18. Rats were separately enclosed in metabolic cages and urine specimens were collected at the bottom of the cages in suitable-sized funnels with plastic perforated discs to retain fecal matter, then collected in beakers and centrifuged for 10 min at about 3,000 rpm to eliminate insoluble materials. The supernatants were transferred into other clean and dry tubes and stored at −20 °C until analysis.

### Measurement of urine total proteins

It was done as showed by Nishi & Elin (1985) utilizing urinary protein assay kit [obtained from Chondrex, Inc. 2607-151st place NE Redmond, WA 98052, USA].

### Blood sampling

Blood specimens were gained from retro-orbital venous plexus under ether anesthesia at GD21 after overnight fasting, then serum was separated by the centrifugation at 3000 rpm for 20 min and kept at (−20 °C) until used.

### Serum analysis

#### Measurement of serum creatinine

This was done as showed by Schirmeister, Willmann & Kieffr (1964) by utilizing Biodiagnostic kit for calorimetric determination of serum creatinine concentration (Biodiagnositc Company, Dokki, Giza, Egypt).

#### Measurement of serum urea

was done as showed by Fawcett & Coctt (1960) utilizing Biodiagnostic kit for calorimetric determination of serum urea concentration (from (Biodiagnositc Company, Dokki, Giza, Egypt).

#### Measurement of Serum Endothelin-1 level (ET-1)

was done as showed by Khraibi, Heublein & Knox (1993) utilizing rat ET-1 ELISA Kit (Phoenix Pharmaceutical Inc., Burlingame, CA).

#### Measurement of serum NO

Serum NO was estimated as nitrite, a NO metabolite, by detecting NO_3_ reduction to NO_2_ by nitrate reductase (sigma), then NO_2_^−^ levels (nitrite levels) were measured using the calorimetric Griess Reaction (Ignarro et al., 1993).

#### Measurement of serum IL-6 level

Measurement was done as shown by Engvall & Perlmann (1971), utilizing rat IL-6 ELISA Kit (from RayBiotech.com).

#### Measurement of serum malondialdhyde (MDA) level

Measurement was down as shown by Satoh (1978) utilizing Biodiagnostic kit (Biodiagnostic company, Dokki, Giza, Egypt).

### Histopathological examination

After collecting blood, rats were sacrificed by cervical dislocation under mild ether anesthesia. kidneys were excised and swilled in ice-cold normal saline (4 °C) to remove blood cells and dried with filter paper then were kept in 10% buffered formalin - saline for at least one week. Then, the fixed tissues were processed habitually, embedded in paraffin. Serial sections from tissue embedded-parrafin blocks were cut at thickness 3-5um, then stained with hematoxylin and eosin (H&E) to study the general microscopic features by routine light microscope (Drury & Wallington, 1980).

### Statistical analysis

The statistical analysis was done concerning 10 rats from each group. Data were given as mean ± SD. SPSS version 19 was utilized for performing the statistical analysis. ANOVA followed by LSD post hoc test was carried out to compare means of the different groups. *P* value <0.05 was considered to be statistically significant for all statistical tests done.

### Sample size calculation

Assuming that serum MDA levels in PE group versus PE+APLN group were  0.55 ± 0.1 versus 0.44 ± 0.1 µmol/l. At confidence level 95% and power 80%, total sample size is 30 rats. We increased sample size to 12 in every group to overcome the risk of rats death during the experiment.Sample size is calculated by Open epi software program.

## Results

This study showed that L-NAME promoted significant increases in mean BP, total urine protein level, urea, creatinine, ET-1, IL-6, and MDA (*p* < 0.001), but decreased serum NO level (*p* < 0.001) in the preeclampsia group when compared to normal pregnant group (Figs. 1–8).

**Figure 1:**
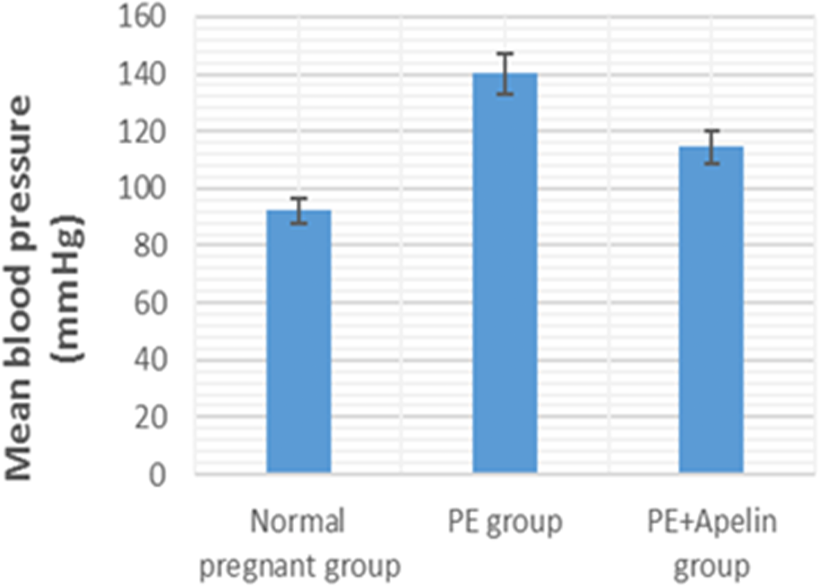
Mean arterial BP in the studied groups. Significant (*P* < 0.001) versus group 1 (normal group). Significant (*P* < 0.001) versus group 2 (PE group).

**Figure 2:**
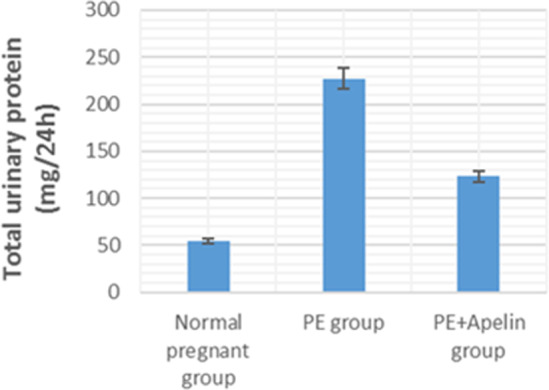
Total urine proteins in the studied groups. Significant (*P* < 0.001) versus group 1 (normal group). ignificant (*P* < 0.001) versus group 2 (PE group).

**Figure 3:**
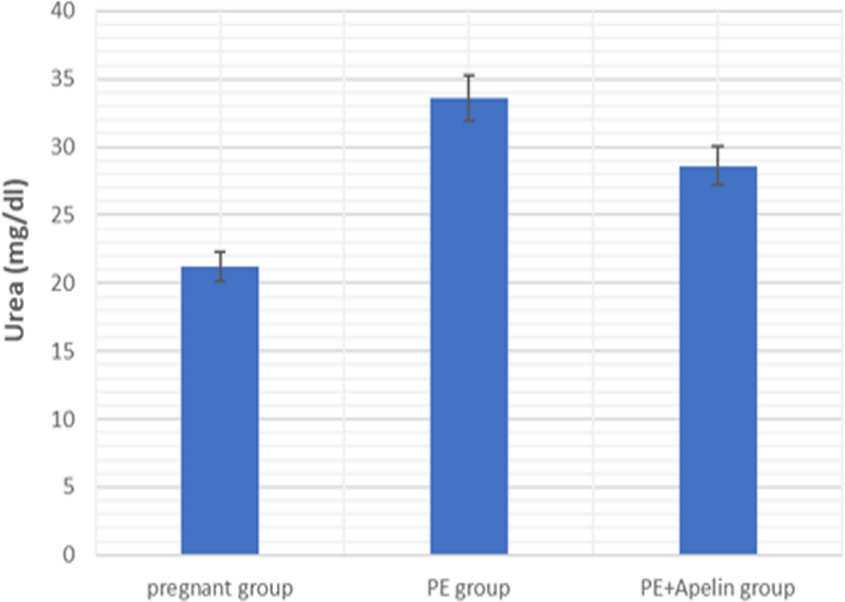
Serum urea in the studied groups. Significant (*P* < 0.001) versus group 1 (normal group). Significant (*P* < 0.001) versus group 2 (PE group).

**Figure 4:**
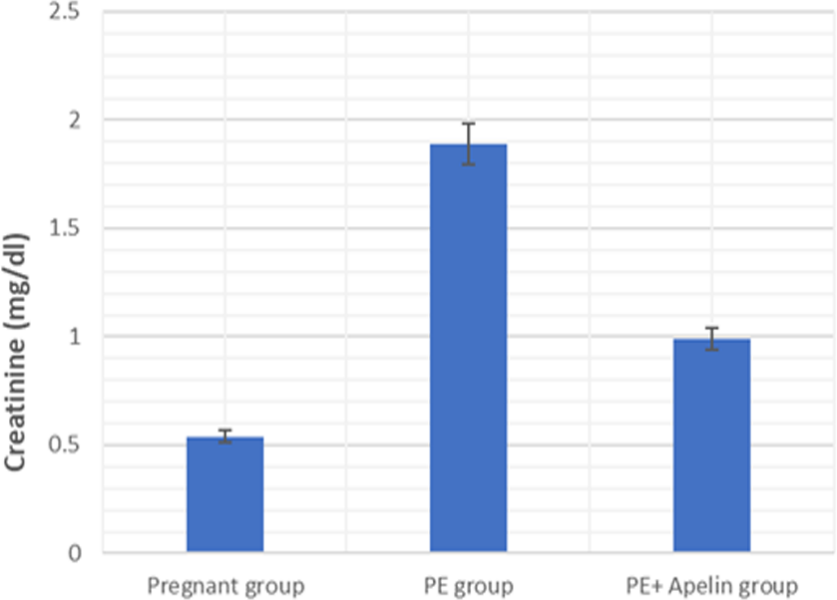
Serum creatinine in the studied groups. Significant (*P* < 0.001) versus group 1 (normal group). Significant (*P* < 0.001) versus group 2 (PE group).

**Figure 5:**
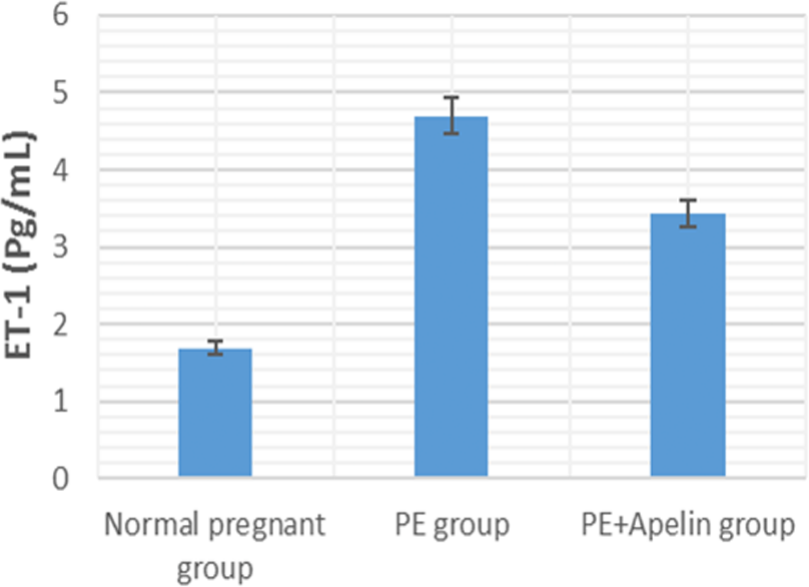
Serum ET-1 in the studied groups. Significant (*P* < 0.001) versus group 1 (normal group). Significant (*P* < 0.001) versus group 2 (PE group).

**Figure 6:**
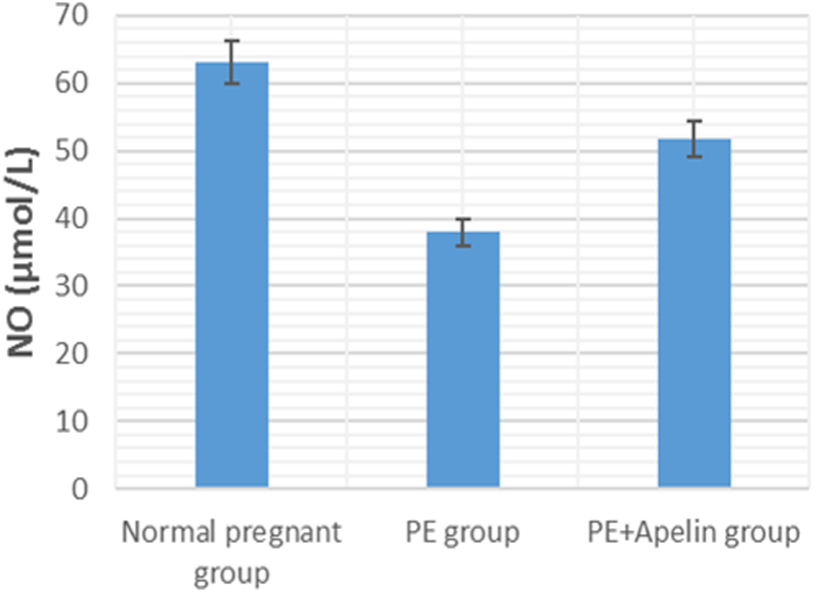
Serum IL-6 in the studied groups. Significant (*P* < 0.001) versus group 1 (normal group). Significant (*P* < 0.001) versus group 2 (PE group).

**Figure 7:**
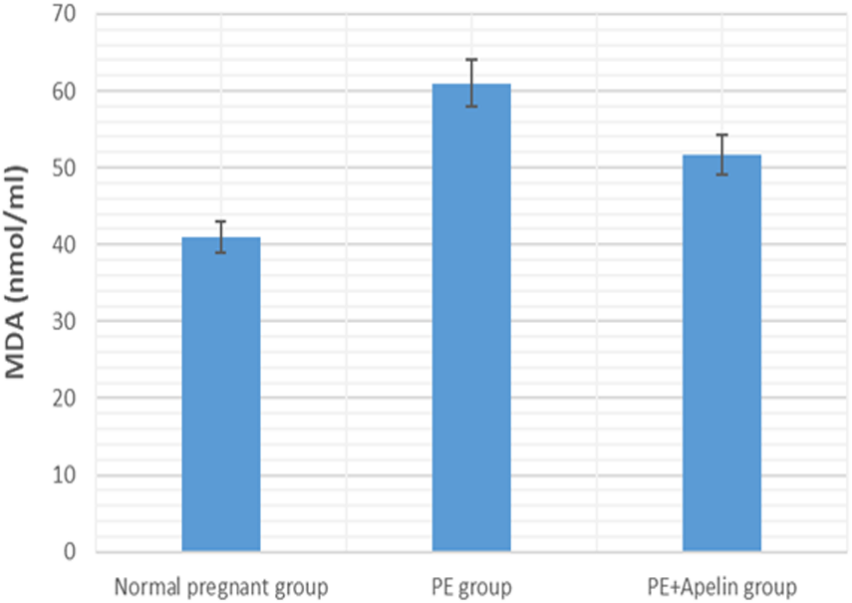
Serum MDA in the studied groups. Significant (*P* < 0.001) versus group 1 (normal group). Significant (*P* < 0.001) versus group 2 (PE group).

**Figure 8:**
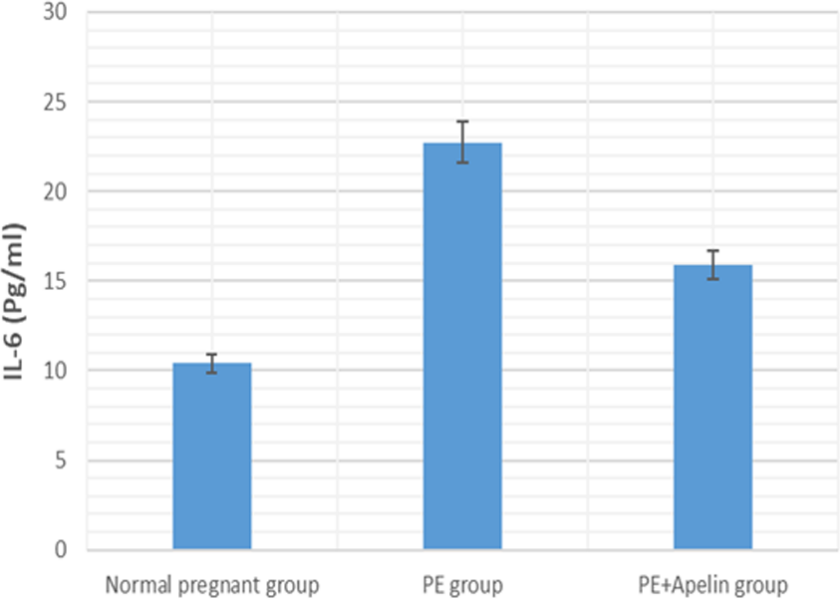
Serum IL-6 in the studied groups. Significant (*P* < 0.001) versus group 1 (normal group). Significant (*P* < 0.001) versus group 2 (PE group).

However, it was showed that apelin administration caused a significant reduction in mean arterial blood pressure, urine protein level, serum urea, creatinine, ET-1, IL-6, and MDA (*p* < 0.001), and significantly increased serum NO level (*p* < 0.001) in the treated group in contrast to untreated pre-eclampsia group (Figs. 1–8).

**Figure 9:**
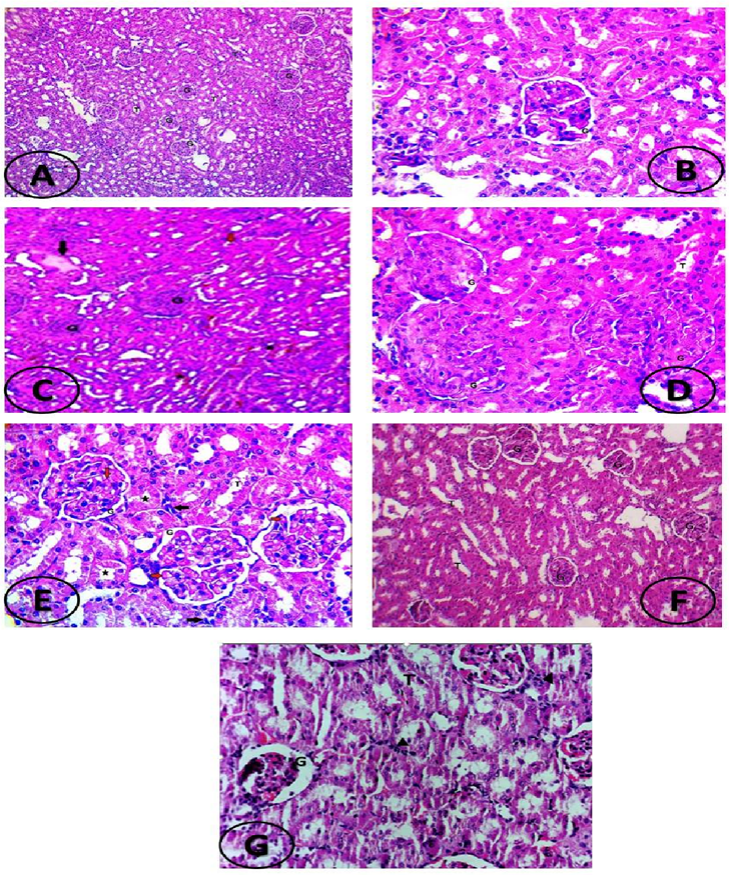
(A–G) Histopathological examination of kidney section. (A) Photomicrograph of kidney tissue of normal control group showing normal kidney histology: normal glomeruli (G) and normal tubules (T). (H&E X100). (B) Photomicrograph of kidney tissue of normal control group showing normal kidney histology: normal glomeruli (G) and tubules (T). (H&E X400). (C) Photomicrograph of kidney tissue of preeclampsia rat showing glomerular hypercellularity (endotheliosis) compressing the Bowman’s space, transudate accumulation (black arrow) in some tubules, cast formation (red arrows) in others, focal areas of hemorrhage in the intersitium (around asterisks); G, glomeruli; T, tubules (H&E X100). (D) Photomicrograph of kidney tissue of preeclampsia rat showing glomerular hypercellularity (endotheliosis) compressing the Bowman’s space; G, glomeruli; T, tubules (H&E X400). (E) Photomicrograph of kidney tissue of preeclampsia rat showing microthrombi formation (red arrows) inside the capillary lumens, degeneration in the tubular epithelium (asterisk) and inflammatory infiltrate in the interstitium (black arrows)(H&E X400). (F) Photomicrograph of kidney tissue of apelin treated PE rat showing mild change in kidney histology: normal glomeruli with mild focal degenerative changes in tubules ; G, glomeruli; T, tubules. (H&E X100). (G) Photomicrograph of kidney tissue of apelin treated PE rat showing mild congested renal vasculature, normal glomeruli, intact Bowman’s space with mild focal degenerative changes in tubules and mild leucocytes infiltration (arrowheads); G, glomeruli; T, tubules (H&E X400).

Also, histopathological evaluation in this study (as shown in Fig. 9) exhibited histopathological signs of kidney damage in the form of glomerular endotheliosis, transudate and cast accumulation in renal tubules, capillary microthrombi formation and inflammatory infiltration with hemorrhagic areas in in the interstitial tissue in the L-NAME induced preeclamptic group (Figs. 9C–9E). On the other hand, all these manifestations were improved by apelin administration in apelin-13 treated preeclamptic group (Figs. 9F and 9G).

## Discussion

PE is a multi-systemic disorder that belongs to pregnancy-related hypertensive disturbance. One of the nitric oxide synthase (NOS) inhibitor named N-nitro-L-arginine-methyl-ester (L-NAME) is used to induce a pre-eclampsia rat model because of the identity between clinical features in L-NAME-treated rats and preeclamptic women including hypertension and renal abnormalities (Ma, Sun & Yang, 2010).

Due to apelin functions on angiogenesis, vasodilation, and immune modulation, it could perform an important role in the treatment and suppression of pre-eclampsia related complications including renal complications.

The present study demonstrated that rats handled with L-NAME showed elevated blood pressure, urinary protein excretion and increased serum urea and creatinine concentrations together with histopathological signs of kidney affection (glomerular endotheliosis, transudate accumulation in some tubules and cast formation in others, together with focal areas of hemorrhage and inflammatory infiltration in the intersitium and microthrombi formation in some capillary lumens). These results are like functional, biochemical and histopathological changes that were demonstrated by other studies (Talebianpoor et al., 2017; Shu et al., 2018; Zuo & Jiang, 2020).

PE outputs, partially, from improper placentation and trophoblast invasion that causes placental ischemia and hypoxic conditions during pregnancy. Placental ischemia, in turn, promotes disproportionate emission of anti-angiogenic agents [as soluble fms-like tyrosine kinase 1 (sFlt-1) and soluble endoglin (sEng)] and pro-angiogenic agents [as placental growth factor (PLGF) and vascular endothelial growth factor (VEGF)], stimulated inflammatory cells releasing autoantibodies and inflammatory cytokines and increased oxidative stress which contribute significantly to generalized maternal endothelial dysfunctions in various organ beds, leading to hypertension, renal endotheliosis and blood coagulation (Gathiram & Moodley, 2016). Endothelial dysfunction, reduced vasodilation, elevated peripheral resistance and the renal disturbances (glomeruloendotheliosis, decreased glomeruli filtration and proteinuria) are all hallmarks of preeclampsia (Erlandsson et al., 2016).

However, treatment with apelin-13 rescued the elevated blood pressure and proteinuria, reduced serum urea and creatinine concentrations and attenuated the kidney histopathological changes observed in L-NAME- induced preeclamptic rat model. These outcomes argued that administration of apelin ameliorated the impairment in nephritic function induced by L-NAME. In accordance with these results, apelin administration has been showed reduction in blood pressure and proteinuria in the preeclampsia models that are created by lowered uterine perfusion pressure (Wang et al., 2017). This amelioration may be, in part, elucidated to the amelioration of renal hemo-dynamics as apelin had been shown to promote vasorelaxation of angiotensin II pre-constricted efferent and afferent arterioles (Hus-Citharel et al., 2008)

Increased progesterone, angiotensinogen (produced from liver by estrogen), renin (produced by extrarenal origins; the ovaries and decidua) and aldosterone levels during normal pregnancy may perform a role in elevating glomerular filtration rate through pregnancy (Hussein & Lafayette, 2014a; Hussein & Lafayette, 2014b). Vasodilation, nonetheless, occurs throughout pregnancy in spite of the elevated rennin–angiotensin–aldosterone system because of several factors such as progesterone and (VEGF)-mediated prostacyclins that enhance resistance to angiotensin II. Also, the existence of AT-1 receptors in a monomeric status in normal pregnancy makes it less sensitive (Granger et al., 2006; Gilbert, Babcock & Granger, 2007).

However, Placental ischemia may contribute to vasoconstriction, elevation of blood pressure, proteinuria and endotheliosis through antiangiogenic agents and autoantibodies which bind to the AT1 receptors and increased angiotensin II sensitivity (Burke et al., 2016). ET1 is a strong vasoconstrictor and has been found experimentally to intermediate the hypertension that is produced by sFLT- 1 and AT1- autoantibodies (George & Granger, 2011).

Our study also observed significantly elevated serum ET-1 level in L-NAME preeclamptic rats compared to control pregnant ones. however, apelin administration significantly reversed the upregulation of serum ET-1 levels. Several stimuli such as growth factors, cytokines, hypoxia, free radicals and ET-1 itself have been notified to stimulate the release of endothelial ET-1. As ET-1 causes oxidative stress that might result in elevated manufacturing of sFlt-1 in the placenta in PE, thus a vicious circle develops that increases ET-1 levels and consequently affects blood pressure and kidney function (Saleh et al., 2016). the ameliorating effect of apelin on ET-1 level may be due to its ameliorative effect on inflammatory cytokines and oxidative stress shown below and could also explain the improvement in kidney function in the treated preeclamptic rats.

Moreover, NO production and excretion by endothelial cells have a significant function in vascular tension regulation and relaxation in pregnancy and promotes the increased blood flow and fetal growth throughout pregnancy (Eckman et al., 2012). Chronic suppression of NO production elevates the blood pressure in a volume-dependent way (Vechoropoulos et al., 2014). The present study observed decreased circulating NO level following treatment with L-NAME in preeclamptic rats compared with that of control ones. On the other hand, administration of apelin-13 reversed the decrease of NO levels in the serum of treated preeclamptic rats. Wang et al. (2017) also stated that the treatment with apelin remarkably improved the endothelial nitric oxide synthase (eNOS) expression in the placenta and in the serum and proposed that reconditioning of the eNOS/NO pathway might be engaged in the ameliorative effects of apelin on preeclampsia. Antifibrotic actions of apelin by the apelin/APJ/Akt/eNOS pathway that oppose Ang II have also been notified in unilateral ureteral ligation- induced renal fibrosis (Nishida et al., 2012).

Furthermore, throughout pre-eclampsia evolution, the emission of diffusible agents as ROS, lipid peroxidation products and inflammatory cytokines from the placenta into the maternal circulation causes systemic endothelial damage (Aouache et al., 2018).

Our results have demonstrated that L-NAME- induced preeclampsia like rat model showed oxidative stress and inflammatory changes notified by elevated serum IL-6 and MDA levels. Similar findings were also noted by Yang et al. (2019). However, the increased MDA levels, and IL-6 in pre-eclamptic rats were declined by administration of apelin. Apelin has also been found to exert reno-protective effects in diabetic nephropathy and ischemia reperfusion by repressing inflammatory response, ROS generation and apoptosis (Day, Cavaglieri & Feliers, 2013) and (Zhang et al., 2020).

Apelin-13 had the possibility to pharmacologically protect versus inflammation and oxidative stress throughout activating the 5′AMP-activated protein kinase / glycogen synthase kinase 3*β* / nuclear factor erythroid 2-related factor 2 (Nrf2) pathway (Duan et al., 2019). Nrf2 is a transcriptional factor that intermediates the antioxidant response. Normally, it is attached to its chaperone; Kelch-like ECH-linked protein 1 (Keap1) and remained in the cytoplasm, and this association enhances its degradation by some proteasomes. Under oxidative stress conditions, Nrf2 separates from Keap1, translocates into the nucleus, attaches to antioxidant response elements (AREs), and upregulates antioxidants and anti-inflammatory modulators formation (de Haan, 2011).

Taking the present results with each other, it can be concluded that apelin treatment can exert reno-protective effects on L-NAME-induced preeclampsia in rats as indicated by improved blood pressure, proteinuria, serum urea and creatinine levels and ameliorated renal histopathologic changes.

The probable mechanisms involved include increasing nitric oxide and decreasing ET-1 bioavailability and inhibiting ROS, lipid peroxidation and inflammation in maternal circulation and renal tissues.

##  Supplemental Information

10.7717/peerj.11110/supp-1 Supplemental Information 1 ARRIVE 2.0 Checklist

10.7717/peerj.11110/supp-2 Supplemental Information 2 Supplemental data

## Additional Information and Declarations

### Data Availability

The following information was supplied regarding data availability: Raw data are available in the Supplemental File. The histopathological images of the experimental work are located in Fig. 9.
